# A Randomized Trial of Insulin Glargine plus Oral Hypoglycemic Agents versus Continuous Subcutaneous Insulin Infusion to Treat Newly Diagnosed Type 2 Diabetes

**DOI:** 10.1155/2018/2791584

**Published:** 2018-10-21

**Authors:** Shuo Lin, Mu Chen, Wanling Chen, Keyi Lin, Panwei Mu, Bilian Zhu, Wen Xu, Manman Wang, Jianping Weng, Longyi Zeng

**Affiliations:** ^1^Department of Endocrinology, The Third Affiliated Hospital of Sun Yat-sen University, No. 600, Tianhe Lu, Guangzhou 510630, China; ^2^Respiratory Department, The Sixth Affiliated Hospital of Sun Yat-sen University, Guangzhou 510000, China; ^3^Department of Endocrinology, The First Affiliated Hospital of Guangzhou Medical University, Guangzhou 510000, China

## Abstract

**Aims:**

Basal insulin plus oral hypoglycemic agents (OHAs) has not been investigated for early intensive antihyperglycemic treatment in people with newly diagnosed type 2 diabetes. This study is aimed at comparing the short-term (over a period of 12 days) effects of basal insulin glargine plus OHAs and continuous subcutaneous insulin infusion (CSII) on glycemic control and beta-cell function in this setting.

**Methods:**

An open-label parallel-group study. Newly diagnosed hospitalized patients with type 2 diabetes and fasting plasma glucose (FPG) ≥11.1 mmol/L or glycated hemoglobin (HbA1c) ≥9% (75 mmol/mol) were randomized to CSII or insulin glargine in combination with metformin and gliclazide. The primary outcome measure was the mean amplitude of glycemic excursions (MAGE), and secondary endpoints included time to reach glycemic control target (FPG < 7 mmol/L and 2-hour postprandial plasma glucose < 10 mmol/L), markers of *β*-cell function, and hypoglycemia.

**Results:**

Subjects in the CSII (*n* = 35) and basal insulin plus OHA (*n* = 33) groups had a similar significant reduction from baseline to end of treatment in glycated albumin (−6.44 ± 3.23% and− 6.42 ± 3.56%, *P* = 0.970). Groups A and B have comparable time to glycemic control (3.6 ± 1.2 days and 4.0 ± 1.4 days), MAGE (3.40 ± 1.40 mmol/L vs. 3.16 ± 1.38 mmol/L; *p* = 0.484), and 24-hour mean blood glucose (7.49 ± 0.96 mmol/L vs. 7.02 ± 1.03 mmol/L). Changes in the C-peptide reactivity index, the secretory unit of islet in transplantation index, and insulin secretion-sensitivity index-2 indicated a greater *β*-cell function improvement with basal insulin plus OHAs versus CSII.

**Conclusions:**

Short-term insulin glargine plus OHAs may be an alternative to CSII for initial intensive therapy in people with newly diagnosed type 2 diabetes.

## 1. Introduction

The progressive nature of type 2 diabetes mellitus is ascribed to a vicious cycle of increasing insulin resistance and progressive pancreatic islet *β*-cell dysfunction [1, 2], caused by the toxic effects of hyperglycemia and gluco-lipotoxicity [3]. Substantial evidences showed that in people with type 2 diabetes, nearly 50% of *β*-cell function is lost by the time of diagnosis, and this loss continues over time irrespective of lifestyle and pharmacological intervention [4]. Recent studies have suggested that early intensive glucose-lowering therapies are effective for managing blood glucose levels and sustaining *β*-cell function and have the long-term benefit of reducing the development of both micro- and macrovascular complications [5–7]. Of these intensive regimens, insulin-based protocols, such as continuous subcutaneous insulin infusion (CSII) or multiple daily injections (MDI), are highly effective at reducing hyperglycemia in patients with poorly controlled type 2 diabetes [8–11]. A meta-analysis of individuals receiving intensive hyperglycemia-reducing regimens showed a significant increase in *β*-cell function and a decrease in insulin resistance, with 46.3% of patients in remission after 12 months without the use of medication and relying solely on lifestyle modification to control blood glucose levels [12]. Two to five weeks of CSII can reduce glucotoxicity, ameliorate insulin resistance, and promote recovery of *β*-cell function in subjects with newly diagnosed type 2 diabetes [13, 14].

However, CSII has shortcomings, with high cost, inconvenience, and relatively complex equipment limiting its uptake by people with type 2 diabetes. Moreover, the need for frequent glucose monitoring during intensive short-term CSII makes implementation in the out-patient setting relatively impractical [15]. Similarly, the MDI approach has limitations such as the need for multiple injections and difficulties with implementation in the out-patient setting. In contrast, basal insulin plus oral hypoglycemic agents (OHAs) has advantages over CSII and MDI including the possibility for convenient once-daily injections [16, 17]. For people with type 2 diabetes poorly controlled by metformin, early addition of the long-acting insulin analogue insulin glargine leads to effective glycemic control and improved *β*-cell function [18, 19]. Indeed, both the American Diabetes Association (ADA) and European Association for the Study of Diabetes (EASD) advocate such regimens for patients with type 2 diabetes who have severe hyperglycemia [20].

In newly diagnosed individuals with type 2 diabetes, short-term use of insulin glargine monotherapy showed noninferior efficacy when compared with CSII in terms of fasting glycemic control and recovery of *β*-cell function. However, postprandial glycemic excursions were less well controlled with insulin glargine versus CSII [21]. Basal insulin plus OHAs may be expected to exert similar or better glycemic control over both fasting and postprandial glucose levels, with improved *β*-cell function. However, while previous studies have investigated and compared CSII, MDI, OHAs, or insulin glargine monotherapy as short-term intensive treatment for people recently diagnosed with type 2 diabetes, basal insulin plus OHAs has not been investigated. This report describes a randomized open-label parallel-group trial of short-term (over a period of 12 days) intensive hypoglycemic therapy with basal insulin (glargine) plus OHAs, compared with CSII treatment, in patients with newly diagnosed type 2 diabetes mellitus, which is aimed at assessing the acute effects of these treatment approaches on glycemic control and *β*-cell function.

## 2. Methods

### 2.1. Patients

Participants aged 25–70 years old with newly diagnosed type 2 diabetes mellitus (according to the 1999 WHO diagnostic criteria [22]) with fasting plasma glucose (FPG) ≥11.1 mmol/L or HbA1c ≥ 9% (75 mmol/mol), who were hospitalized between November 2014 and November 2015, and had not previously received medication for diabetes were eligible for inclusion. Patients were excluded if they tested positive for autoimmune antibodies to islet cells, were pregnant or breast-feeding, had acute or chronic complications of diabetes, had chronic heart failure, had impaired renal function or clinically evident hepatic disease, acute infection, took drugs which may affect glucose metabolism (corticosteroids, antipsychotics, immunosuppressive drugs, etc.), or would not confirm that they would comply with the study protocol. All patients gave written informed consent for enrollment into the study, which was approved by the ethics committee of the Third Affiliated Hospital of Sun Yat-sen University. This study was registered at ClinicalTrials.gov (NCT02526810).

### 2.2. Study Design and Treatment

This was an open-label, single center, randomized, parallel-group, noninferiority trial conducted at The Third Affiliated Hospital of Sun Yat-sen University (Guangzhou, Guangdong, China). All subjects were hospitalized throughout the study and were addressed in an educational training. Patients were provided with balanced diets for diabetes, recommended taking 30 minutes of physical exercise after meals, and educated on diabetes self-management. Patients were randomly assigned (1 : 1) to either CSII monotherapy (group A) or basal insulin plus OHA (group B). Randomization was performed using sealed and opaque envelopes arranged in a computer-generated random order, prepared by specific staff and opened sequentially to determine the patients' treatment assignments. Patients in group A received a fast-acting insulin analog (insulin Aspart, Novo Nordisk, Bagsvaerd, Denmark) as basal medication and prandial insulin administered via an insulin pump (Paradigm 722, Medtronic, Minneapolis, MN). Patients in group B received insulin glargine (Lantus, Sanofi-Aventis, Paris, France) injection once daily at bedtime, oral metformin (Glucophage) 0.5 g twice daily, and gliclazide (Diamicron MR) 60 mg each morning. Total initial insulin doses were 0.5–0.8 IU/kg for the CSII group (including basal and bolus insulin, 1 : 1) and 0.2 IU/kg for the basal insulin plus OHA group. Insulin doses were titrated every day by doctors using a glycemic target defined as fasting capillary blood glucose < 7 mmol/L and 2-hour postprandial blood glucose < 10 mmol/L. The basal insulin dosage was given by using the titration algorithm based on FPG (Supplementary Materials (available here)). Metformin was added gradually to a maximum dose of 1 g twice daily unless intolerable, in which case a lower dose could be administered. Gliclazide was added to 90 mg or 120 mg by doctors while 2-hour postprandial blood glucose ≥ 10 mmol/L with fasting capillary blood glucose < 7 mmol/L.

### 2.3. Endpoints

The primary study endpoint was mean amplitude of glycemic excursions (MAGE) after reaching glycemic target. Secondary endpoints included time to reach glycemic target (fasting capillary blood glucose < 7 mmol/L and 2-hour postprandial plasma glucose (PPG) <10 mmol/L), change of HbA1c, fasting plasma glucose (FPG), PPG, glycated albumin, low-density lipoprotein cholesterol (LDL-C), high-density lipoprotein cholesterol (HDL-C) and triglycerides, assessment of glucose excursions, pre- and postprandial mean blood glucose level (MBG), levels of insulin secretion, C-peptide levels, assessment of *β*-cell function, and incidence of hypoglycemia.

### 2.4. Assessments

Demographic data were recorded on enrollment. A standard mixed meal tolerance test was performed in the fasted state before the start and at the end of the study with 0, 30, and 120-minute blood samples collected. The meal comprised 562 kcal with 51.2% carbohydrates, 33.4% lipids, and 15.4% protein and was consumed over 15 minutes. During the study, capillary blood glucose was monitored at least seven times a day (before, and 2 hours after, each meal and at bedtime) using a blood glucometer (OneTouch Ultra, LifeScan, Milpitas, CA). Normoglycemia was confirmed for 1 to 3 days after the glycemic target was achieved, and then, a continuous glucose monitoring system (Medtronic Paradigm, 722) was used to monitor glycemic variability for 72 hours before study treatment was stopped. After stopping the treatment in group A (for more than 12 hours) and group B (metformin and gliclazide for more than 24 hours, glargine for more than 36 hours), a standard mixed meal tolerance test was performed and blood samples were collected in each subject as well as baseline.

Venous blood was drawn for measurement of FPG and PPG, HbA1c, glycated albumin, lipid profile, fasting and postprandial C-peptide and insulin, and routine clinical laboratory analyses. Plasma glucose was measured using the glucose oxidase method. Plasma triglycerides, total cholesterol, HDL-C, and LDL-C were measured using an automatic oxide test. HbA1c was measured using the Bio-Rad (Hercules, CA) Variant HbA1c assay. Plasma insulin and C-peptide levels were measured with a chemiluminescence enzyme-linked immunosorbent assay (Bioekon Inc., Beijing, China). Proinsulin was measured using the Total Human Proinsulin ELISA kit (EMD Millipore, USA). Laboratory tests were performed in the central clinical laboratory of the Third Affiliated Hospital of Sun Yat-sen University.

The area under the curve (AUC) for insulin, glucose, and C-peptide during the standard mixed meal tolerance test was calculated using the trapezoidal rule. A homeostasis model assessment of insulin resistance (HOMA-IR) and beta-cell function (HOMA-*β*) was used to estimate insulin sensitivity and *β*-cell function [23], and the Matsuda index was used to evaluate whole body insulin sensitivity [24]. The secretory unit of islet in transplantation (SUIT) index and C-peptide reactivity index (CPI) were used to estimate *β*-cell function [25, 26].

Early-phase insulin secretion in response to glucose was assessed using the insulinogenic index (△insulin_0.5h_/△glucose_0.5h_), calculated as the incremental insulin response (0–30 minutes) divided by the incremental glucose response (0–30 minutes) [27]. The insulin secretion-sensitivity index-2 (ISSI-2) was also used as a validated measurement of *β*-cell function ([AUC insulin/AUC glucose] × Matsuda index) [9, 28]. The ratio of proinsulin to immunoreactive insulin (PI/IRI) was also calculated as a marker of *β*-cell function.

Glucose fluctuation parameters such as MAGE, standard deviation of blood glucose levels (SDBG), MBG, proportion of the time in hyperglycemia (>7.8 mmol/L), proportion of the time in hypoglycemia (<3.9 mmol/L), 1-hour fasting MBG, and 3-hour postprandial MBG, were assessed by continuous glucose monitoring [29]. The MAGE was manually calculated as the mean of differences between consecutive peaks and nadirs, with excursions < 1 standard deviation excluded. Change in BMI after treatment, the time and medication dose required to reach glycemic targets, and hypoglycemia occurrence were recorded. Hypoglycemia was defined as blood glucose < 3.9 mmol/L with or without clinical symptoms. A severe hypoglycemic episode was defined as a hypoglycemic event requiring the assistance of another person.

### 2.5. Statistical Analyses

Based on previous research, this noninferiority study was powered to detect an intergroup difference in MAGE of 0.3 mmol/L assuming a standard deviation of 0.5 mmol/L, type 1 error of 0.05, and type 2 error of 0.2. Using these assumptions, a sample size of 34.4 patients in each group was calculated, giving a total enrolment target of 70 patients.

Descriptive variables were compared between groups using an unpaired Student's *t*-test for parametric data and the Mann–Whitney *U* test for nonparametric data. Repeated measurements were assessed using a sample-paired Student's *t*-test and Wilcoxon matched pair test for nonparametric data. To compare the treatment effect on MAGE between the two groups, the analysis of covariance (ANCOVA) was used, with treat groups as the fixed main effect, and mean capillary blood glucose (seven-point) at baseline as the covariate. Pearson's (parametric data) or Spearman's (nonparametric data) tests were used to detect correlations between variables. All statistical procedures were processed using SPSS software for Windows version 19.0. (SPSS Inc., Chicago, IL). A two-sided *p* value of <0.05 was considered statistically significant.

## 3. Results

### 3.1. Patients

A total of 79 patients with newly diagnosed type 2 diabetes mellitus were assessed for suitability for the study, and 71 were randomized to treatment (group A, *n* = 36; group B, *n* = 35) (Supplementary Figure 1). One patient randomized to group A left the study early for financial reasons and two in Group B left early due to gout (*n* = 1) and personal circumstance (*n* = 1). The final analysis population therefore included 68 patients (35 in group A and 33 in group B).

Baseline demographic characteristics of study subjects were comparable between the two groups, with similar mean age (group A, 47.6 ± 10.9 years; group B, 49.0 ± 9.9 years), proportion of males (group A, 71.4%; group B, 69.7%), and BMI (group A, 25.4 ± 3.7 kg/m^2^; group B, 25.3 ± 2.8 kg/m^2^). Laboratory parameters at baseline were also generally comparable between the two groups, with the exception of triglyceride levels which were higher for subjects in group B (1.51 ± 0.70 vs. 2.19 ± 1.23 mmol/L, *p* = 0.006) (Table 1). No differences were observed between baseline values of glucose assessment parameters (HbA1c, glycated albumin, FPG, and PPG), PI/IRI, or indices of *β*-cell secretion (CPI, SUIT, △INS_0.5h_/△GLU_0.5h_, and ISSI-2) (Tables 1 and 2). Baseline HOMA index was also comparable between the two groups. There was no significant difference in mean capillary blood glucose (seven-point/day) at baseline between group A (13.6 ± 2.5 mmol/L) and group B (14.2 ± 3.4 mmol/L) (*P* = 0.389).

### 3.2. Glycemic Control and Lipid Profiles

The time to achieve glycemic target (3.6 ± 1.2 days vs. 4.0 ± 1.4 days) and duration of treatment (10.5 ± 1.7 days vs. 10.6 ± 1.8 days) were similar for subjects in group A and group B (*P* > 0.05). A similar significant reduction from baseline to end of treatment in glycated albumin (−6.44 ± 3.23% and − 6.42 ± 3.56%) and HbA1c levels (−0.94 ± 0.50% [−10.3 mmol/mol] and − 0.80 ± 0.35% [−8.7 mmol/mol]) was observed for groups A and B, respectively (Table 1). After reaching glycemic targets, the basal and bolus insulin doses of group A were 20.3 ± 5.6 U and 25.5 ± 5.9 U, respectively, while the median dose of metformin and gliclazide and mean insulin dose in group B were 1.0 g/day, 60 mg/day, and 17.9 ± 6.7 U, respectively. Twenty-four patients (72.7%) took gliclazide 60 mg daily when the glucose target was reached. Only one patient (3.0%) in group B added gliclazide up to 120 mg. Eight patients (24.2%) reduced to 30 mg daily by doctors for concern of hypoglycemia. At the end of the study, the basal and bolus insulin doses of group A were 17.7 ± 6.5 U and 25.6 ± 7.7 U, respectively, while the median dose of metformin and gliclazide and mean insulin dose in group B were 1.5 g/day, 60 mg/day, and 11.2 ± 5.2 U, respectively. There was significant difference in mean basal insulin doses between group A and group B (*P* < 0.01).

**Figure 1:**
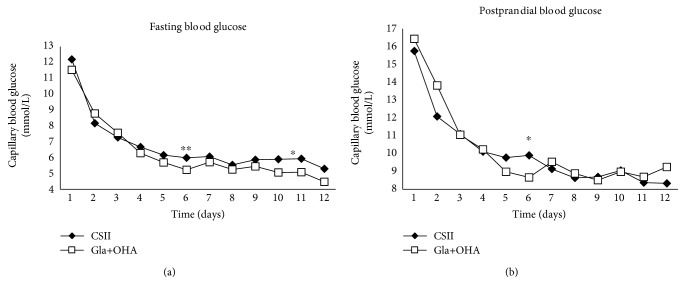
Changes in the levels of fasting blood glucose (a) and postprandial blood glucose (b) in subjects receiving continuous subcutaneous insulin infusion (group A) or basal insulin glargine plus oral hyperglycemic agent (group B) ^∗^ *p* < 0.05, ^∗∗^ *p* < 0.01.

Both treatment regimens decreased FPG and 2-hour PPG over the 12-day treatment period (Figures 1(a) and 1(b)). Mean FPG levels were significantly lower in group B versus group A at day 6 (*p* < 0.001) and day 10 (*p* < 0.05), and PPG was also significantly lower for this group at day 6 (*p* < 0.05). A smaller reduction in mean FPG (−5.85 ± 2.92 mmol/L vs. −7.41 ± 3.41 mmol/L, *p* = 0.046) and 2-hour PPG (−7.19 ± 4.18 mmol/L vs. −9.78 ± 4.94 mmol/L, *p* = 0.022) levels was observed for subjects in group A versus group B.

Lipid profiles showed a similar significant reduction in LDL-C and total cholesterol levels in both treatment groups (Table 1). For HDL-C and triglycerides, a significant decrease was only seen for group B (*p* < 0.01), although no significant differences in the change in lipid levels were observed between the treatment groups.

### 3.3. Glycemic Excursions

Similar levels of glycemic excursions were observed after treatment in CSII versus basal insulin plus OHAs for controlling (Table 3); MAGE was 3.40 ± 1.40 mmol/L for group A and 3.16 ± 1.38 mmol/L for group B (*p* = 0.484). After adjusted with mean capillary blood glucose (seven-point/day) at baseline (by using ANCOVA), the estimated MAGE was 3.44 ± 0.23 mmol/L for group A and 3.12 ± 0.24 mmol/L for group B, respectively (*p* = 0.352). Other measures of glucose fluctuations including SDBG, frequency of glycemic excursion (FGE), mean of daily differences (MODD), and AUC < 3.9 values were also comparable between treatment groups, although the AUC > 7.8 was significantly lower for group B (*p* = 0.003). Continuous glucose monitoring showed that the preprandial 1-hour MBG level was significantly lower for patients in group B before breakfast (*p* = 0.004), lunch (*p* = 0.047), and dinner (*p* = 0.002), and the postprandial 3-hour MBG level after lunch (*p* = 0.021) was also lower for group B. However, the difference between groups was not significant for the 3-hour postprandial MBG level after breakfast and dinner.

### 3.4. Insulin Resistance and *β*-Cell Function

Levels of insulin and C-peptide were higher after treatment (*p* < 0.01) in both groups (Supplementary Figures 2A and 2B). A greater increase in fasting plasma insulin, 30-minute postprandial plasma insulin, and C-peptide levels was seen for group B compared with group A. The AUCs for both insulin and C-peptide during a mixed meal tolerance test were significantly elevated after treatment (both *p* < 0.01), with no significant difference between groups (*p* = 0.230 and *p* = 0.360, respectively). HOMA-IR decreased significantly for both groups (*p* < 0.01), with no significant differences observed between the treatment groups before or after insulin treatment (Table 2).

All *β*-cell secretion indices improved significantly over the 12-day study period, including HOMA-*β*, CPI, SUIT, △INS_0.5h_/△GLU_0.5h_, and ISSI-2 (all *p* < 0.01), indicating that *β*-cell function was substantially restored for both treatment groups. Furthermore, the recovery of rapid-phase insulin secretion was comparable for both treatment groups, as shown by the change in △INS_0.5h_/△GLU_0.5h_ (*p* = 0.06) by the end of treatment. The PI/IRI values decreased significantly (both *p* < 0.01) after treatment for both treatment groups, with no significant intergroup difference (*p* = 0.432).

### 3.5. Effects on BMI and Hypoglycemia

No differences were observed between groups A and B for change in body weight (−0.16 ± 0.89 kg and 0.07 ± 0.72 kg, respectively, *p* = 0.658) or BMI (−0.05 ± 0.32 kg/m^2^ and −0.02 ± 0.28 kg/m^2^, respectively, *p* = 0.723) during the study. In group A, 19 of 35 patients (54.3%) experienced a total of 27 hypoglycemic events, and no patients experienced severe hypoglycemia; in group B, 22 of 33 patients (66.7%) experienced a total of 42 hypoglycemic events, and no patients experienced severe hypoglycemia. The frequency of hypoglycemia did not differ between groups.

## 4. Discussion

To the authors' knowledge, this is the first randomized trial to compare the effect of short-term intensive therapy with basal insulin plus OHAs versus CSII in people with newly diagnosed type 2 diabetes mellitus. The results of the study show that individuals treated with both basal insulin plus OHAs and CSII achieve optimal glycemic control within around 4 days. A similar significant reduction from baseline to end of treatment in glycated albumin and HbA1c levels was observed. After reaching the blood glucose target, glucose fluctuations as determined by MAGE, SDBG, FGE, and MODD were similar in both treatment groups. Furthermore, *β*-cell function was improved with short-term intensive treatment, irrespective of whether basal insulin plus OHAs or CSII was used. These results demonstrate that basal insulin plus OHA therapy may be an effective short-term intensive therapy for relieving hyperglycemia and restoring *β*-cell function.

In the present study, glycemic excursions, as measured by MAGE, SDBG, and MBG, were similarly controlled with basal insulin plus OHAs and CSII. A previous study of basal insulin monotherapy versus CSII for early intensive treatment of type 2 diabetes reported greater glycemic excursions with basal insulin compared with CSII [21]. This suggests that the addition of OHAs to basal insulin leads to tighter control of blood glucose compared with basal insulin alone. Indeed, this is not surprising given that basal insulin mainly controls FPG, and the addition of metformin plus gliclazide would be expected to exert greater control over postprandial blood glucose levels. This finding is important given that PPG excursions have been associated with myocardial infarction and cardiovascular mortality [30]. In addition, tighter control of blood glucose levels is associated with greater reductions in HbA1c and lower incidence of hyper- or hypoglycemia.

Previous studies have shown that administering 2 weeks of CSII to newly diagnosed patients with type 2 diabetes resulted in almost a 1% decrease in HbA1c and an 8% decrease in glycated albumin, while the time taken to achieve glycemic targets was around 3 to 5 days [13, 21]. The results of the present study are comparable regarding the time taken to reach glycemic target for both CSII and basal insulin plus OHAs. This finding indicates that basal insulin glargine plus OHAs is an effective treatment for managing hyperglycemia in newly diagnosed patients with type 2 diabetes mellitus. A major benefit of insulin glargine plus OHA therapy is the single daily insulin injection. Patient adherence to therapy is a key factor for achieving glycemic control, and the difficulty in managing multiple injections and multiple daily glucose measurements represents a major barrier to compliance [31]. The importance of adherence to medication has been evaluated in a number of prospective and retrospective studies [32, 33], which show that glycemic control rates improve progressively with better medication compliance. In this regard, insulin glargine plus OHA treatment may be expected to result in better compliance than requiring patients to administer twice-daily insulin.

Previous studies reported that rapid correction of hyperglycemia, or so-called glucotoxicity, by short-term intensive therapies, has substantial benefits in terms of promoting islet cell functional recovery through “*β*-cell rest” [12, 13, 21]. In this study, CPI, SUIT, and ISSI-2 were used to measure endogenous insulin secretion; these methods correlate well with the glucose clamp test and are widely used in studies assessing *β*-cell function. Data from the present study suggest that basal insulin plus OHA therapy rapidly corrects hyperglycemia and also confers *β*-cell function benefits, possibly by eliminating glucotoxicity and providing a *β*-cell rest period. In addition, several studies have been conducted to assess the effect of neutralising glucotoxicity through short-term (2–3 weeks) intensive antihyperglycemic treatment on improving insulin resistance. A 2013 meta-analysis of such studies reported a reduction in HOMA-IR of −0.57 (95% CI; −0.84 to −0.29) [12]. This is in close agreement with the reduction in HOMA-IR achieved with CSII over 12 days in the present study (−0.56), although individuals who received basal insulin plus OHA achieved a slightly lower reduction (−0.33). However, it is known that remission rates differ for patients receiving total exogenous insulin replacement by CSII or MDI and those receiving agents that promote endogenous insulin secretion such as oral sulfonylureas, indicating that besides glucose toxicity there are likely to be other factors that affect *β*-cell function [13]. As such, further studies are required to compare remission rates following intensive short-term CSII or basal insulin plus OHAs.

The recovery of first-phase insulin secretion and decreased PI/IRI ratio has been confirmed for both short-term intensive insulin and oral medication therapies [13]. However, there has been controversy over the effects of sulfonylurea on the PI/IRI ratio, with some studies showing an increase and others a decrease [34–36]. Interestingly, the current study showed that the combination of basal insulin with gliclazide and metformin was able to restore early-phase secretory function and markedly decrease the PI/IRI ratio, indicating improvements in both quantitative and qualitative insulin production.

The primary limitation of this study was that only the short-term benefits of intensive insulin treatment were investigated, which does not allow for long-term observations. Second, the study was conducted at a single center and had small sample size. Third, MAGE could not be adjusted for baseline differences between the groups because of the absence of baseline measurement of MAGE. However, there was no statistical difference between mean seven-point blood glucose at baseline in two groups (*P* > 0.05). After adjusted for mean blood glucose at baseline, the estimated MAGE was similar in two groups (*p* = 0.352). These results suggested that glycemic excursions were comparable between two groups. Despite these limitations, the demonstration that basal plus OHA therapy is able to restore glycemic control and improve *β*-cell function warrants further investigation.

In conclusion, short-term intensive therapy with basal insulin plus OHAs showed comparable benefits to CSII in terms of overall glycemic control and improvement in *β*-cell function in newly diagnosed patients with type 2 diabetes mellitus and is a possible option for treating newly diagnosed patients with type 2 diabetes.

## Figures and Tables

**Table 1:** Glycemic and lipid assessment parameters from baseline to the end of study treatment with continuous subcutaneous insulin infusion (group A) or basal insulin glargine plus oral hyperglycemic agent (group B).

Clinical characteristic^†^	Group A (*n* = 35)	Group B (*n* = 33)	*P* value
Age	47.6 ± 10.9	49.0 ± 9.9	0.590
Male (*n*, %)	25, 71.4	23, 69.7	0.876
BMI (kg/m^2^)	25.4 ± 3.7	25.3 ± 2.8	0.839
WC (cm)	90.9 ± 8.0	90.8 ± 8.2	0.937
Baseline HbA1c (%) [mmol/mol]	11.38 ± 2.00 [100.9]	11.17 ± 2.00 [98.6]	0.671
△HbA1c (%) [mmol/mol]	−0.94 ± 0.50^‡^ [−10.3]	−0.80 ± 0.35^‡^ [−8.7]	0.190
Baseline glycated albumin (%)	32.70 ± 7.00	31.74 ± 9.04	0.625
△Glycated albumin (%)	−6.44 ± 3.23^‡^	−6.42 ± 3.56^‡^	0.970
Baseline FPG (mmol/L)	12.72 ± 2.87	12.41 ± 3.33	0.681
△FPG (mmol/L)	−5.85 ± 2.92^‡^	−7.41 ± 3.41^‡^	0.046
Baseline 2-hour PPG (mmol/L)	21.73 ± 3.93	20.79 ± 4.93	0.389
△2-hour PPG (mmol/L)	−7.19 ± 4.18^‡^	−9.78 ± 4.94^‡^	0.022
Baseline LDL-C (mmol/L)	3.44 ± 1.24	3.48 ± 1.06	0.905
△LDL-C (mmol/L)	−0.76 ± 0.95^‡^	−1.04 ± 0.91^‡^	0.221
Baseline total cholesterol (mmol/L)	5.21 ± 1.27	5.63 ± 1.21	0.165
△Total cholesterol (mmol/L)	−0.95 ± 0.97^‡^	−1.43 ± 1.02^‡^	0.054
Baseline HDL-C (mmol/L)	1.09 ± 0.25	1.17 ± 0.40	0.289
△HDL-C (mmol/L)	−0.03 ± 0.16	−0.10 ± 0.15^‡^	0.087
Baseline triglycerides (mmol/L)	1.51 ± 0.70	2.19 ± 1.23	0.006
△Triglycerides (mmol/L)	−0.10 ± 0.64	−0.50 ± 1.08^‡^	0.062

^†^Data are mean (standard deviation) unless otherwise stated; ^‡^significant change from baseline (*p* < 0.05). △: change from baseline; BMI: body mass index; WC: waist circumference; FPG: fasting plasma glucose; PPG: postprandial plasma glucose; HbA1c: glycated hemoglobin; HDL-C: high-density lipoprotein cholesterol; LDL-C: low-density lipoprotein cholesterol.

**Table 2:** Changes in *β*-cell function and insulin sensitivity from baseline to day 12 in patients treated with continuous subcutaneous insulin infusion (group A) or basal insulin glargine plus oral hyperglycemic agent (group B).

Indices^†^	Group A (*n* = 35)	Group B (*n* = 33)	*P* value for group comparison
Baseline AUC insulin (h·mU/L)	35.83 ± 24.99	36.34 ± 23.21	0.932
△AUC (h·mU/L)^‡^	19.59 (6.50–31.19)	27.24 (14.49–51.83)	0.060
Baseline AUC C-peptide (h·nmol/L)	1.50 ± 0.70	1.46 ± 0.62	0.826
△AUC C-peptide (h·nmol/L)^‡^	0.45 (−0.04–0.72)^§^	0.62 (0.21–1.05)^§^	0.323
Baseline LnHOMA-IR	1.43 ± 0.48	1.30 ± 0.62	0.355
△LnHOMA-IR	−0.56 ± 0.63^§^	−0.33 ± 0.53^§^	0.113
Baseline LnHOMA-*β*	2.85 ± 0.77	2.81 ± 0.90	0.828
△LnHOMA-*β*	1.06 ± 0.82^§^	2.36 ± 0.98^§^	0.000
Baseline CPI	0.70 ± 0.43	0.72 ± 0.39	0.867
△CPI	0.34 ± 0.32^§^	0.74 ± 0.49^§^	0.000
Baseline SUIT	15.76 ± 12.36	16.22 ± 10.39	0.870
△SUIT	18.01 ± 12.31^§^	63.99 ± 47.70^§^	0.000
Baseline IGI 30 minutes	1.91 ± 2.19	1.56 ± 1.66	0.464
△IGI 30 minutes	1.33 ± 3.00^§^	2.64 ± 2.64^§^	0.06
Baseline ISSI-2^‡^	49.76 (38.39–75.68)	63.90 (36.51–113.25)	0.568
△ISSI-2^‡^	136.30 (109.22–184.83)^§^	212.51 (168.25–268.80)^§^	0.000
Baseline PI/IRI %^‡^	36.0 (24.9–64.6)	37.9 ± 24.5	0.401
△PI/IRI%^‡†^	−21.8 (−35.6–4.9)^§^	−26.5 ± 24.6^§^	0.432

^†^Data are mean (standard deviation) unless otherwise stated; ^‡^median (range); ^§^significant change from baseline (*p* < 0.05). △: change from baseline; AUC: area under the curve; ISSI: insulin secretion sensitivity index; HOMA-*β*: homeostatic model assessment of *β*-cell function; CPI: C-peptide reactivity index; SUIT: the secretory unit of islet in transplantation index; IGI: insulinogenic index; PI/IRI: ratio of proinsulin to immunoreactive insulin; HOMA-IR: homeostatic model assessment or insulin resistance.

**Table 3:** Glycemic excursion parameters at the end of treatment with continuous subcutaneous insulin infusion (group A) or basal insulin glargine plus oral hyperglycemic agent (group B).

Indices^†^	Group A (*n* = 35)	Group B (*n* = 33)	*P* value for group comparison
MAGE (mmol/L)	3.40 ± 1.40	3.16 ± 1.38	0.484
SDBG	1.41 ± 0.53	1.21 ± 0.53	0.119
FGE^†^	3 (3–4)	3 (3–4)	0.908
MODD	1.64 ± 0.55	1.54 ± 0.75	0.520
AUC < 3.9^‡^	0.00 (0.00–0.02)	0.00 (0.00–0.00)	0.142
AUC > 7.8	0.46 ± 0.37	0.24 ± 0.18	0.003
1-hour prebreakfast MBG (mmol/L)	7.11 ± 1.20	6.26 ± 1.17	0.004
1-hour prelunch MBG (mmol/L)	7.24 ± 1.71	6.46 ± 1.44	0.047
1-hour predinner MBG (mmol/L)	7.78 ± 1.58	6.69 ± 1.19	0.002
3-hour after breakfast MBG (mmol/L)	7.56 ± 1.23	7.18 ± 1.23	0.207
3-hour after lunch MBG (mmol/L)	7.84 ± 1.64	6.98 ± 1.36	0.021
3-hour after dinner MBG (mmol/L)	8.08 ± 1.43	7.52 ± 1.41	0.111
24-hour MBG (mmol/L)	7.49 ± 0.96	7.02 ± 1.03	0.056

^†^Data are mean (standard deviation) unless otherwise stated. ^‡^Median (range) AUC > 7.8 mmol/L, area under the curve above 7.8 mmol/L; AUC < 3.9 mmol/L, area under the curve below 3.9 mmol/L. MBG: mean blood glucose; SDBG: standard deviation of blood glucose; MAGE: mean amplitude of glycemic excursions; FGE: frequency of glycemic excursion; MODD: mean of daily differences.

## Data Availability

The data used to support the findings of this study are available from the corresponding author upon request.

## Supplementary Materials

Supplementary Materials Supplementary Figure 1: patients' flow diagram. Supplementary Figure 2: changes in insulin (A) and C-peptide (B) levels during a standard meal in the two study groups, at the end of treatment with continuous subcutaneous insulin infusion (group A) or basal insulin glargine plus oral hyperglycemic agent (group B). Supplementary: the titration algorithm of basal insulin in group B.
