# Supplementary material for: A Randomized Trial of Insulin Glargine plus Oral Hypoglycemic Agents versus Continuous Subcutaneous Insulin Infusion to Treat Newly Diagnosed Type 2 Diabetes
Source: J Diabetes Res. 2018 Oct 21;2018:2791584. doi: 10.1155/2018/2791584 (PMC6215559; doi:10.1155/2018/2791584)
Supplement: Supplementary Materials — Supplementary Figure 1: patients' flow diagram. Supplementary Figure 2: changes in insulin (A) and C-peptide (B) levels during a standard meal in the two study groups, at the end of treatment with continuous subcutaneous insulin infusion (group A) or basal insulin glargine plus oral hyperglycemic agent (group B). Supplementary: the titration algorithm of basal insulin in group B. [file 2791584.f1.docx]

Supplementary Figure 1. Patients flow diagram


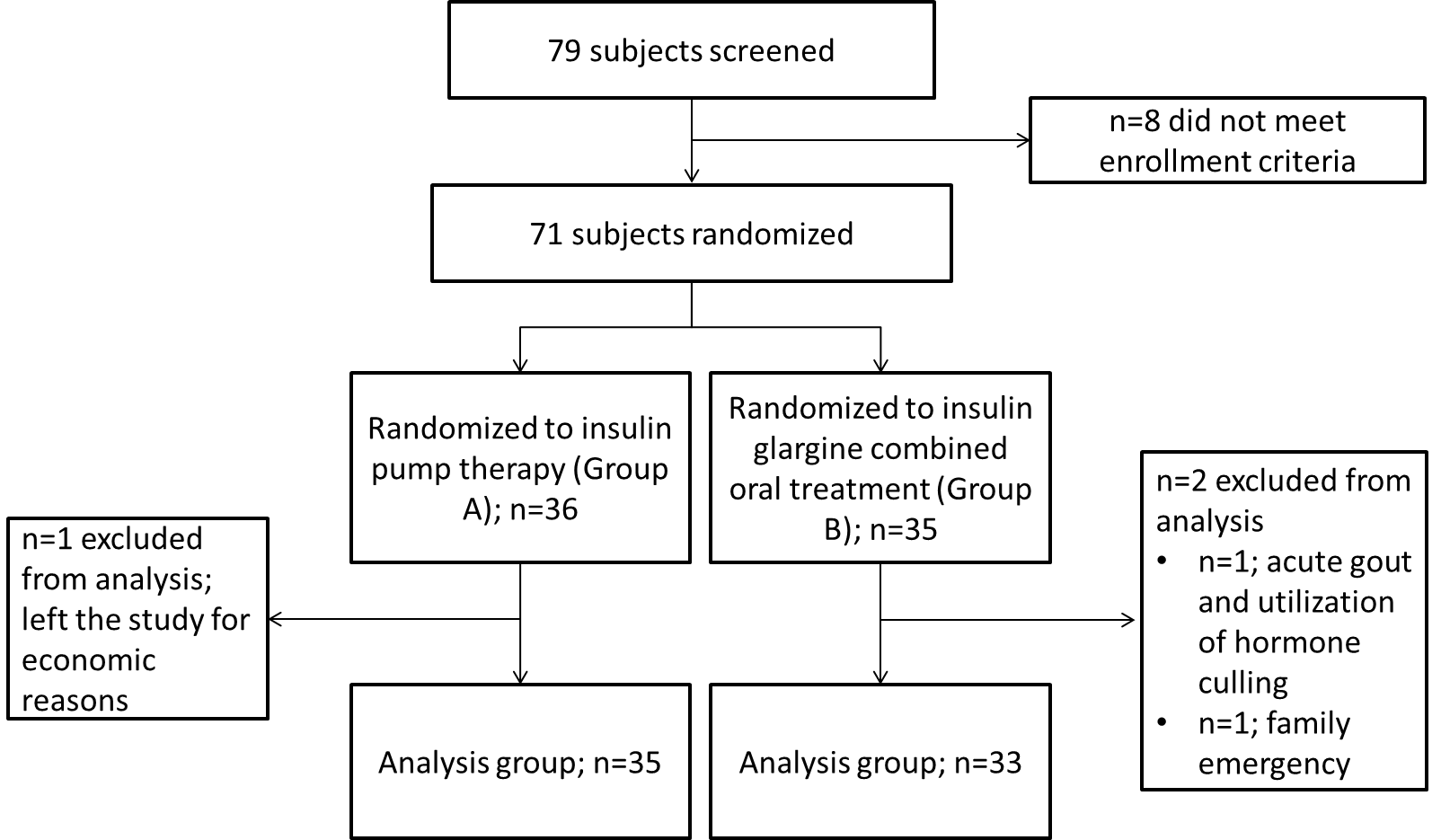


Supplementary Figure 2. Changes in insulin (A) and C-peptide (B) levels during a standard meal in the two study groups, at the end of treatment with continuous subcutaneous insulin infusion (Group A) or basal insulin glargine plus oral hyperglycemic agent (Group B)

**

**

*

*p<0.05, **p<0.01.

**Supplementary. the titration algorithm of basal insulin in group B**

The insulin dosage was given using the following titration algorithm based on FPG : FPG ≥ 6.1–7.7 mmol/L, increase by 2 U/day; FPG >7.7–10.0 mmol/L, increase by 4 U/day; FPG >10.0 mmol/L, increase by 6 U/day; and FPG<3.9mmol/L, reduced by 10-20%.
